# Natural Abilities and Mental Requirements in the Study and Practice of Dentistry

**Published:** 1883-03

**Authors:** Morrell A. Webb

**Affiliations:** Marengo, Ill.


### ARTICLE III.

### *Natural Abilities and Mental Attainments Requisite to the Study and Practice of Dentistry.*

BY DR. MORRELL A. WEBB, MARENGO, ILL.

For a man to engage in any occupation, or profession with profit to himself and patrons, he must possess some knowledge of his own natural tendencies, also of his abilities in the direction of the field of labor upon which he expects to enter, and his physical abilities should be taken into account as well.

I wish to present a few thoughts in relation to what is necessary to know about one's self before entering upon the study and practice of dentistry. For a man to possess himself of the required knowledge relating to himself, he must study the works of God in all their different departments, and, for convenience, I shall separate the necessary field of attainment into some ot the most important branches:

1. A man must believe in God; he must have a saving knowledge of him, and in so doing he will live an unselfish life, working for the cause of the great truths, not looking for reward so much in this life as in the next; and in so doing he will lead a life of honesty, and have the implicit confidence of those for whom he may labor. He will possess a consciousness that he has done his best for his patients, and thus be much surer of living a long life here, and being able to enter into the glorious life beyond.

2. The dentist must have a thorough education, and thus be able to understand the duties he may be called upon to perform; to understand the causes of the diseases he may treat; to know the composition and origin of the metals and materials which he daily manipulates, and the composition of the medicinal preparations that he administers. And to become acquainted with these things it is necessary for him to study the following sciences:

1. Astronomy, because it teaches him from whence comes the light that so beautifies our world, the size and magnitude of the body which supplies us with heat from its everlasting fires, and, too, that he may become acquainted with those laws which are so wonderfully and perfectly kept, that the movements of the planets do not vary a hair's breadth in a million years, and that he may realize that the laws that govern the construction and destiny of his own body, are no less wonderful and perfect, and that, did man obey the laws of his being, there would be no need of the profession of dentistry.

2. He must acquaint himself with geology, as it teaches him the formation of the earth, of what it is composed, that it holds in its bosom the metals and substances which he is daily using; and that a few of its elements enter into a combination which forms so natural a substitute for the teeth, porcelain, and also that he may be able to present to his mind a clear idea of the composition and appearances of the different periods in the earth's formation, that he may know from whence comes that fuel which enables us to turn nature's darkness into day, and at the same time to drive away the chill winter blast from our hearthstones.

3. He must study chemistry for the purpose of understanding those laws which govern, and the properties of those substances used in the operating room, and laboratory; and the forces necessary to change their forms and nature. He will learn the composition of air, water, metals, vegetable and animal substance, and all other material forms connected with the earth. He will understand the laws which govern the action of metals as used by him; and, in fact a thorough knowledge of chemistry is very essential to a proper consideration and investigation of the Science of Dentistry.

4. He must acquaint himself with physiology that he may know the action and uses of the organs and parts of of the human body, more especially, that he may see the relation one organ bears to another; that he may see how the improper working of one may lead to the disorder of another; how we are made to realize that a part of the organism is below its normal standard of action; how those white glistening fibres, although microscopical in size, act as the telegraph wires to warn us of approaching danger from external sources, or internal disease; and he will thus learn of the formation and development of the different organs of the body.

5. The dentist must study the science of philosophy to become posted in the laws of natural forces, and of mechanics, in the kinds of electricity and its generation and magnetism as they are connected with natural forces, and with the relation which they hold to man and especially to the dentist.

6. Anatomy next requires thorough mastery to be able to understand the construction and development of the organs of the body, that he may know of what substances the teeth are composed, how they may be altered in density or appearance, what muscles of the head and face act in the process of mastication, at what age the teeth are severally erupted, how they are connected one with the other by nerves and blood vessels, and thence with the general organism, and how, and where they are situated in their relation to the face. In fact, a thorough course in anatomy is desired.

7. The dentist should be posted in matters relating to history that he may converse with intelligence about past events, either as relating to his own calling, or matters generally, that he may see the profit of thorough study and investigation, by the great changes that have taken place in theories and beliefs which once held sway among the most intelligent. He will, by the study of the history of the science of dentistry, perceive how great changes have been wrought in the methods and modes of practice, and be stimulated to continue the good work of separating the dross from the pure, and placing present theory either on broad foundation of facts, or else burying it deep from sight forever.

8. The dentist should be literary in his tastes, that he may study the thoughts, and profit by the expressions of the same, of the best minds of the age, and of ancient times. He should love poetry because he will thus learn of the beauties of nature, at the same time he enjoys the beauties of language, and because his soul will be elevated to nobler and greater deeds, looking towards the elevation of his less favored brethern. By reading subjects relating to his calling, as expressed by the most intelligent minds in its ranks, he will be better able to give his mite of information to help swell the tide of knowledge that bears the profession onward to still greater perfection.

And there are other and numerous branches of knowledge that require study and research. And all taken together help the man who contemplates the practice of dentistry, to better understand himself.

A man, to be a really great man in this profession, must possess genius; he must have the eye of the mechanic, the perceptive faculties of the artist, and the greatest steadinese of hand and strength of muscle.

He must have the eye of the mechanic, too see beforehand the completed operation whatever it may be, and the laws necessary to obey to make it successful, how to shape the cavity, how to leave the margins, how hard to bring pressure to bear upon the walls of the cavity, how to work in unison with the laws governing the metals or substances which he uses in filling a tooth.

He must have the perfection of the artist to see his work clearly in the mind, that he may know where and how each and every movement of the instrument tends to the completion of his filling to the beautiful and original contour of the tooth, avoiding a disobedience of the law, of course, which says "that a thing is only as strong as its weakest part," thus avoiding building a filling out of proportion to the support of its foundations, thereby avoiding (as is often the case) the breaking away of the walls and ruination of the work.

The dentist must be well stocked with nervous power, that he can stand a continued strain, with but little fatigue; and he must be possessed of the recuperative powers necessary to return his exhausted energies to their normal standard. The nervous strain that the dentist undergoes is a fruitful cause of the breaking down of the physical powers, not only because he has to stand with steadiness and use his instruments with the greatest precision, but because he does not have the rest needed to regain that strength already lost, thus differing from the average physician who is seldom called upon to perform more than one surgical operation a day, and that usually of short duration, thus avoiding that continued strain which the dentist necessarily bears.

I have given only a synopsis, as it were, of those studies and natural abilities requisite to the greatest good in the man, and dentist: and that the more we know of the facts and principles of the world, and, in fact, those which underlie the working of this mighty fabric of the universe, and the more we know of those thoughts advanced by the intelligence of man, (enlightened by research and investigation,) regarding cause and effect, and the nearer we get to the truth of the object of our being, and the great love manifested towards us by the Creator, the nearer we come to a perfect self-knowledge, and the greater, better, and more perfect will be the works of our lives, and those with whom we associate will be the better for our living, and in the end it can be said of each of us: "Well done, good and faithful servant."—*Ohio State Journal of Dental Science.*

